# Shear stress effects on epididymal epithelial cell via primary cilia mechanosensory signaling

**DOI:** 10.1002/jcp.31475

**Published:** 2024-11-07

**Authors:** Sepideh Fakhari, Gabriel Campolina‐Silva, Farnaz Asayesh, Laura Girardet, Marie‐Pier Scott‐Boyer, Arnaud Droit, Denis Soulet, Jesse Greener, Clémence Belleannée

**Affiliations:** ^1^ Department of Obstetrics, Gynecology, and Reproduction, Centre de recherche en Reproduction, Développement et Santé Intergénérationnelle Faculty of Medicine Québec City Quebec Canada; ^2^ Centre de recherche du centre hospitalier universitaire de Québec ‐ Université Laval Québec City Quebec Canada; ^3^ Department of Chemistry Faculty of Science and Engineering Québec City Quebec Canada; ^4^ Proteomics Platform, Québec Genomic Center, Université Laval, CHU de Québec Research Center (CHUL) Québec City Quebec Canada; ^5^ Faculté de pharmacie Université Laval Québec City Quebec Canada

**Keywords:** calcium signaling, epididymis, fluid shear stress, mechanosensor, microfluidic, primary cilia

## Abstract

Shear stress, resulting from fluid flow, is a fundamental mechanical stimulus affecting various cellular functions. The epididymis, essential for sperm maturation, offers a compelling model to study the effects of shear stress on cellular behavior. This organ undergoes extensive proliferation and differentiation until puberty, achieving full functionality as spermatozoa commence their post‐testicular maturation. Although the mechanical tension exerted by testicular fluid is hypothesized to drive epithelial proliferation and differentiation, the precise mechanisms remain unclear. Here we assessed whether the responsiveness of the epididymal cells to shear stress depends on functional primary cilia by combining microfluidic strategies on immortalized epididymal cells, calcium signaling assays, and high‐throughput gene expression analysis. We identified 97 genes overexpressed in response to shear stress, including early growth response (Egr) 2/3, cellular communication network factor (Ccn) 1/2, and Fos proto‐oncogene (Fos). While shear stress triggered a rapid increase of intracellular Ca^2+^, this response was abrogated following the impairment of primary ciliogenesis through pharmacological and siRNA approaches. Overall, our findings provide valuable insights into how mechanical forces influence the development of the male reproductive system, a requisite to sperm maturation.

## Graphical abstract

Influence of shear stress on epididymis epithelial cells through primary cilia mechanosensory signaling. (a) Anatomical illustration of the male reproductive system highlighting the epididymis three main segments: caput, corpus, and cauda. (b) Luminal fluid flow‐induced shear stress within the epididymal duct. (c) Cross‐sectional diagram of the epididymal epithelium showing various cell types including undifferentiated cells, differentiated principal, and clear cells. Primary cilia elongate from the basal body of undifferentiated cells and principal cells. (d) Fluid shear stress‐induced calcium signaling and gene expression in principal cells. Fluid flow induces Ca²⁺ influx through the primary cilium, activating the release of calcium from endoplasmic reticulium (ER) and leading to the modulation of over 100 genes, including flow‐sensitive genes, cilia‐dependent genes, and genes dependent on both flow and the presence of cilia.

## 1 INTRODUCTION

Primary cilia are microtubule‐based organelles that protrude as a solitary antenna from the surface of various cell types in vertebrates (Bloodgood, 2009; Satir, 2017). Extensive investigations have shown that primary cilia function as sensory organelles adept at translating a spectrum of environmental signals, including chemical, and mechanical (Davenport & Yoder, 2005; Praetorius & Spring, 2001; Praetorius, 2015; Spasic & Jacobs, 2017), and electrical signals of the nervous system (Kirschen & Xiong, 2017) into intricate intracellular changes. This unique capacity positions them as key regulators influencing fundamental cellular processes such as proliferation, migration, differentiation, and planar polarity. The significance of primary cilia in physiological processes becomes even more captivating when considering a growing body of evidence suggesting that deformities and dysfunctions in these organelles contribute to a diverse array of human disorders collectively referred to as ciliopathies (Hildebrandt et al., 2011; Reiter & Leroux, 2017).

Primary cilia consist of three main fundamental components: a basal body, originating from the mother centriole of the centrosome; an axoneme, a microtubule‐based core featuring nine doublets of microtubules with no central pair (9 + 0); and the ciliary membrane, which surrounds the axoneme of the primary cilium and has a unique composition rich in specific lipids and proteins (e.g., GPCRs and calcium receptors) (Pazour & Bloodgood, 2008; Satir & Christensen, 2007; Wheway et al., 2018). The primary cilium exhibits cell cycle‐dependent dynamics, as it is disassembled during the G1 and S phases of the cell cycle, coinciding with mitotic activities, and reassembles as cells enter the quiescent G0 phase, reflecting its roles in signaling and sensory functions (Girardet et al., 2019; Mill et al., 2023). The formation and functionality of primary cilia hinge on the intraflagellar transport (IFT) mechanism operating within the axoneme. The dynamic IFT mechanism underlies the intricate structure and functional capabilities of primary cilia (Scholey, 2008).

Over the past decade, there has been a growing interest in investigating the role of primary cilia as mechanosensors, particularly in response to shear stress. Studies in primary cilia‐based mechanotransduction have predominantly concentrated on exploring structural, intracellular, or tissue‐level functional responses to shear stress. An immediate and observable response of primary cilia to fluid shear force is their bending. This bending phenomenon represents a crucial aspect of the mechanosensitive nature of primary cilia and forms a key focus in understanding their response to shear stress (Downs et al., 2014; Rydholm et al., 2010; Schwartz et al., 1997). Cellular mechanosensing through primary cilia is predominantly associated with intraciliary calcium signaling and the subsequent regulation of target genes. Several studies have provided evidence supporting the notion that shear stress triggers a rise in intracellular calcium signaling (Mohammed et al., 2017; Praetorius, 2015; Xu et al., 2007, 2009), resulting in changes in downstream gene expression across various cell types, including vascular endothelial cells (AbouAlaiwi et al., 2009; Boo & Jo, 2003; Zaragoza et al., 2012), liver cells (Masyuk et al., 2006), and bone cells (Kwon et al., 2010; Malone et al., 2007). These investigations have identified specific gene expressions associated with proteins localized in primary cilia, playing pivotal roles in mechanotransduction processes, such as polycystin‐1 (PC1) and polycystin‐2 (PC2) present on the ciliary membrane and that are activated by ciliary deflection (AbouAlaiwi et al., 2009; Kunnen et al., 2018; Miceli et al., 2020; Mohammed et al., 2018). Impairment of primary cilia exposed to shear stress is implicated in conditions such as polycystic kidney disease (Yoder et al., 2002) cystic and fibrotic liver disorders (Masyuk et al., 2008), and abnormalities in bone growth (Nishimura et al., 2012; Temiyasathit et al., 2012; Xiao et al., 2011).

Beyond the liver, bone, and kidney, primary cilia are present in various components of the male reproductive system (Girardet et al., 2019; Vinay et al., 2024), including in the epididymis. The latter is a long tubule attached to the testis via the efferent ductules, playing a role in the control of male fertility through its influence on sperm maturation and storage (Bedford, 1963; Jones, 1999; Orgebin‐Crist, 1967). It has been previously described in rats that at birth, the epididymis is composed of columnar undifferentiated cells that progressively proliferate and differentiate to give rise, before puberty, to principal, basal, and clear cells (Pinel et al., 2019) that are found all along the three main regions of the tubule (i.e. the caput, corpus, and cauda) (Hermo et al., 1992; Sun & Flickinger, 1979). During this period, primary cilia extend from the surface of the epithelium where they enter in contact with the extracellular fluid deriving from the testis. After puberty in mice, while centrioles are strategically positioned at the apical pole of principal cells to form a primary cilium, axonemal extensions are not produced (Bernet et al., 2018). Instead, primary cilia are found exclusively associated with basal cells where they control their stem cell capacity and tissue regeneration through the modulation of the Hedgehog signaling pathway (Girardet et al., 2022). Therefore, it is particularly at the time of epithelial cell differentiation that primary cilia form a privileged interface between epididymal cells and the extracellular fluid flow. It has long been proposed that the first wave of testicular fluid drives epithelial cell differentiation in epididymides (Sun & Flickinger, 1982). While primary cilia are potent cellular sensors responsive to shear stress in most biological tubules, the contribution of this organelle in the control of cellular fate and epididymis development remains unknown. This study aims to evaluate the mechanosensory role of primary in the control of epididymal gene expression and cellular functions.

To do so, we conducted a comprehensive study combining microfluidic imaging with high throughput gene expression profiling on DC2 immortalized epididymal principal cell lines. Following pharmacological and genetic blockage of primary ciliogenesis, we deciphered a calcium‐dependent epididymal cell's response to extracellular shear stress and identified how this physical cue could impact epididymis development. Overall, our study underscores the fundamental importance of primary cilia mechanosensation in the intricate orchestration of epididymal development, providing valuable insights into potential implications for male reproductive physiology.

## 2 MATERIALS AND METHODS

### 2.1 Cell culture

Immortalized distal caput principal cells (DC2) derived from mouse epididymis were generously provided by Marie‐Claire Orgebin‐Crist (Araki et al., 2002). DC2 cells were cultured at 33°C and 5% CO_2_ in Iscove's Modified Dulbecco's Medium (IMDM, 10‐016‐CV, Corning), supplemented with 1 nM dihydrotestosterone (DHT, Fluka), 10% Fetal Bovine Serum (FBS) (A3160702, Gibco), 100 µM MEM nonessential amino acids (11140‐050, Gibco), 1 mM sodium pyruvate (11360‐070, Gibco), and 50 U/m penicillin G (25‐001‐CI, Corning).

### 2.2 Mouse model

A double transgenic mouse model, Tg (CAG‐EGFP/CETN2)34Jgg/KvandJ (ARL13B‐CETN2 tg; Jackson Laboratory stock#027967), was employed to facilitate the endogenous detection of ARL13B, a primary cilia marker, and CENTRIN2, a centriolar protein. Mice were housed and bred in the Specific pathogen‐animal facility at the Centre Hospitalier Universitaire de Quebec Research Center. Ethical approval for the animal studies was obtained from the Institutional Review Board of the Centre Hospitalier Universitaire de Québec (CHUQ; CPAC licenses 16‐050‐4 and 16‐051‐4, C. Belleannée). All procedures were conducted in strict adherence to the ARRIVE guidelines. Epididymis samples were harvested from 2‐week‐old mice (*n* = 3) and fixed in 4% paraformaldehyde (PFA). Following cryopreservation in *30*% *sucrose* solution in phosphate buffer saline (PBS), tissues were embedded in O.C.T. compound (23‐730‐571, Thermo Fisher Scientific) and sectioned at 16‐μm thickness in a cryostat for immunofluorescence assays.

### 2.3 Immunofluorescence staining

Following a 15‐min wash of OCT with PBS, antigen retrieval was conducted in Tris‐EDTA buffer (10 mM, pH 9) for 10 min at 80°C. Subsequently, sections were rehydrated for 15 min in PBS, permeabilized for 6 min with 1% sodium dodecyl sulfate (SDS) and 0.1% Triton X‐100 in PBS, washed in PBS for 5 min, and then blocked for 1 h with 1% BSA and 5% goat serum in PBS. For immunostaining, primary antibodies listed in supplementary Table 1, appropriately diluted in DAKO antibody diluent (S0809, DAKO Corp., Carpinteria), were applied to the tissues and then allowed to incubate overnight in a humid chamber at 4°C. Sections were subjected to two washes with high‐salt PBS (2.7% NaCl) and one wash with PBS for 5 min each. Subsequently, a secondary antibody listed in Supporting Information S1: Table 2 was applied to the tissues at room temperature. For imaging, the slides were mounted with a DAPI‐containing medium (Vectashield, VECTH1200, Vector Laboratories).

For immunofluorescent staining on DC2 cells, coverslips were prepared by coating them with Human plasma Fibronectin at 2% dilution (Millipore, FC010) before cell seeding. Cells were then fixed with 4% PFA for 15 min, followed by two washes with PBS. Subsequently, permeabilization, blocking, and the addition of the primary antibody were carried out, mirroring the procedures outlined for tissue immunofluorescence. After an overnight incubation with the primary antibody, the cells underwent three washes with PBS. Following this, the secondary antibody was applied for 1 h. After additional washes, slides were mounted in Vectashield medium.

### 2.4 Confocal microscopy imaging

Immunostainings of the caput epididymis from the ARL13B‐CETN2 double transgenic model were acquired using a Zeiss LSM800 confocal microscope equipped with Airyscan high‐resolution capability (Zeiss Laboratories). Images were captured with 40× objective lenses (numerical aperture = 2.5–0.6) at room temperature, using optical Z‐sections for each channel. These images were then combined into a single image using the Z‐project tool in ImageJ software.

### 2.5 3D reconstruction

The 3D reconstructions of confocal acquisitions were performed using the image processing toolbox. IMARIS® software (Bitplane, version 7.5.1) was utilized for three‐dimensional reconstruction and animation of confocal image stacks using the Surpass module (Supporting Information S1: Videos 1,2, and 3).

### 2.6 Cell seeding in microfluidic plates and fluid shear stress stimulation

DC2 cells were subjected to biologically relevant shear stress through the induction of laminar fluid flow with a Bioflux 1000z (Fluxion Biosciences Inc.). This comprehensive setup comprises a microfluidic plate, a Bioflux controller capable of delivering a flow shear range spanning from 0.1 to 20 dyne/cm^2^ through an electro‐pneumatic pumping system, and an inverted microscope (ZEISS Axio Observer 7 Illumination) equipped with an sCMOS high‐speed resolution camera (Figure 2a). The experimental procedure was as follows: 20 µL of DC2 cells with a density of 4 million/mL were initially introduced to the inlet wells of microfluidic plates (910‐0047, Fluxion Biosciences) and then seeded into microchannels, pretreated with Hu plasma Fibronectin (Millipore, FC010) at 2% dilution at the first day. The cells were incubated at 33°C overnight. Forty‐eight hours of synchronization was performed on the second and third days by replacing the standard cell culture medium with a low serum medium containing 0.5% FBS. Subsequently, cells were incubated at 33°C overnight. On the final day, cells were subjected to either a static condition or were exposed to fluid shear stress at 1 dyne/cm² for 3 h. To assess the effect of shear stress on epididymal cells, a total of six experimental replicates were analyzed under static control (ST) conditions and six replicates were subjected to fluid shear stress (FL).

To investigate whether the fluid‐shear response in DC2 cells relies on primary cilia, we conducted experiments identical to those described above. This involved 24 h of synchronization and subsequent pharmacological inhibition of cilia formation for 24 h using 30 µM Ciliobrevin D (EMD Millipore, Calbiochem) in DMSO (D8418, Sigma Aldrich). Furthermore, we inhibited primary cilia formation by employing siRNA to knock down IFT88.

### 2.7 siRNA gene silencing

**Figure 1:**
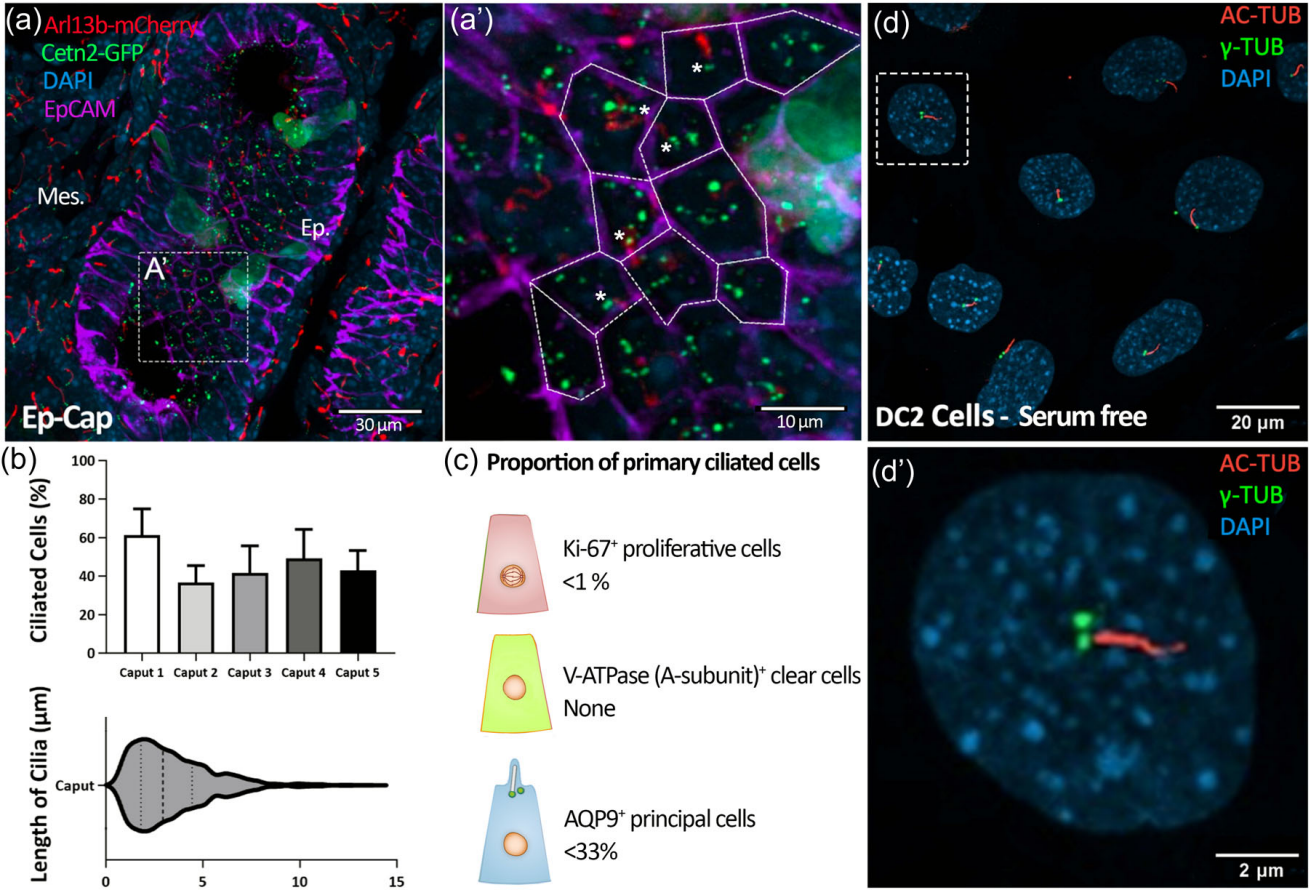
Primary cilia extend from the surface of prepubertal epididymal mouse epithelium and immortalized DC2 cells. (a) The detection of primary cilia was conducted on epididymis sections from 2‐week‐old ARL13b‐CETN2 double‐transgenic mice using confocal microscopy. Epithelial cell adhesion marker EpCAM was used to detect epithelial cell boundaries in the proximal region of the epididymis (Caput, Ep‐Cap). In the inset (A'), Arl13b‐mCherry and Cetn2‐GFP positive primary cilia are detected at the surface of some cells (stars). Mes.: mesenchyme, Ep.:epithelium. (b) The proportion of ciliated cells in the epithelium of caput epididymis and their cilia lengths were determined from *n* = 5 animals. (c) The proportion of proliferative and differentiated cells was assessed from reconstructed confocal microscopy stacks of epididymis sections from 2 weeks old ARL13b‐CETN2 double transgenic mice stained for Ki‐67 proliferation marker, V‐ATPase (subunit A) clear cell marker and AQP9 principal cell marker (see 3D reconstruction supplementary video files). (d) Under serum starvation conditions, immortalized principal cells of the epididymis (DC2 cells) extend primary cilia that are positive for acetylated tubulin (AC‐TUB, axoneme) and gamma‐tubulin (γ‐TUB, basal body).

To circumvent the possible off‐target effects of dynein inhibition, we prevented the formation of primary cilia through the silencing of IFT88, a protein required for ciliogenesis (Taschner et al., 2012). Cells were transfected with 100 nM of a mixture of three small interfering RNAs (siRNAs) targeting different coding sequences of mouse Ift88 (186729; 186730; 186731; Thermo Fisher Scientific) for 24 h. The concentration was selected based on efficiency tests performed with concentrations ranging from 10 to 100 nM (Supporting Information S1: Figure 1). A scrambled siRNA (SC‐37007, Santa Cruz Biotechnologies) was a negative control. Transfection was carried out using Lipofectamine RNAiMAX transfection reagent (13778150, Thermo Fisher Scientific) and Opti‐MEM Reduced Serum Medium (31985070, Thermo Fisher Scientific) following the manufacturer's instructions. The effectiveness of Ift88 silencing was assessed by real‐time PCR, western blot analysis, and quantification of both the number of ciliated cells and the length of cilia. The timeline for experiments assessing the calcium signal to flow is described as follows: on the first day, cells were seeded in the microchannel density of 2 million/mL. On the second day, cells were treated with Ift88 siRNA. On the third and fourth days cells were kept in normal cell culture media and, on the fifth day, media were replaced with low‐serum media. On day six, the cells were utilized for further experimental procedures.

### 2.8 RNA sequencing

RNA extraction was performed on DC2 cells subjected to diverse flow conditions by directly injecting the RLT cell lysis buffer (Quiagen) supplemented with 10% β‐mercaptoethanol (M3148, Sigma Aldrich) into the biofluidic microchannels. Total RNA purification was performed using the RNeasy Mini Kit (Qiagen) according to the manufacturer's protocol. To eliminate potential genomic contamination, incubation with RNase‐free DNase (Qiagen) was performed. The amount of total RNA recovered was quantified using a NanoDrop 1000 microvolume spectrophotometer (Thermo Fisher Scientific). RNA sequencing libraries were prepared using the NEBNext Ultra II Directional RNA library prep kit for Illumina (New England's Biolabs Inc.). Initially, the NEBNext poly(A) (New Englands Biolabs Inc.) was used to purify 150 ng of total RNA, serving as a template for cDNA synthesis by reverse transcriptase with random primers. Adapter ligation was performed, followed by purification with the AxyPrep Mag PCR Clean‐up kit (Axygen, Big Flats) and a PCR enrichment step of 13 cycles to incorporate specific indexed adapters for multiplexing. The quality of the final amplified libraries was examined using a DNA screen tape D1000 on a TapeStation 2200, and quantification was carried out with the QuBit 3.0 fluorometer (Thermo Fisher Scientific). Subsequently, mRNA‐seq libraries with unique indexes were combined in equimolar ratio and subjected to paired‐end 100 PB sequencing on a NovaSeq. 6000 flowcell S2 at the Next‐Generation Sequencing Platform, Genomics Center, CHU de Québec‐Université Laval Research Center, Québec City, Canada. The library's average insert size was 320 bp, and the mean coverage per sample was 25 M paired‐end reads.

Following the acquisition of raw sequencing data in fastq format, their quality was validated through FastQC software (Andrews, 2010). Subsequent preprocessing involved the use of Trimmomatic (Bolger et al., 2014) for the removal of adapter content and over‐represented sequences. Trimmed sequences were then aligned to the mouse transcriptome (Mus_musculus. Ensembl110) using the Kallisto tool (Bray et al., 2016). Normalization procedures were executed utilizing the Relative Log Expression (RLE) method (Anders et al., 2013). Transcript quantifications were converted to gene quantifications using the R‐package Tximport (Soneson et al., 2015). Differentially expressed genes (DEGs) were discerned employing an FDR q‐value < 0.05 and absolute Fold Change > 1.5, facilitated by the R‐package DESeq. 2 (Love et al., 2014). Pathway enrichment analyses, encompassing Gene Ontology Biological Processes (GO: BP), Kyoto Encyclopedia of Genes and Genomes (KEGG), Reactome (REAC), and WikiPathways (WP) databases, were conducted using gene symbols as input. The R package gprofiler2 (Kolberg et al., 2020) was employed, with selected options for analysis including correction_method=fdr and user_threshold=0.05. All computational analyses were executed within the R statistical environment (version 4.3.1). All raw RNA sequencing data have been deposited in the GEO repository under accession number GSE269872.

### 2.9 Gene ontology and pathway analysis

Multiple functional analysis suites, namely DAVID, Metscape, ShinyGO, MouseMine, and STRING, were used to systematically investigate genes influenced by flow‐induced stress. Each suite provided unique insights and perspectives.

### 2.10 Cell proliferation assay

A 5‐Ethynyl‐2′‐deoxyuridine (EdU) incorporation (Click‐iT EdU, C10337, Invitrogen) assay was employed to assess the DC2 cell proliferation rate. Briefly, cells were seeded in microchannels and incubated for 2 h at 33°C to facilitate adherence to the surface. Subsequently, cells in some channels were subjected to shear stress of 1 dyne/cm^2^ for 22 h, while cells in the remaining channels were maintained in a static condition for the same duration. Following this, all cells were treated with 10 µM EdU at 33°C for 1 h. The cells were then fixed, permeabilized, and labeled using the Click‐iT reaction cocktail plus DAPI, following the manufacturer's instructions. Through quantitative analysis, the rate of proliferative cells was calculated by dividing the number of proliferative cells (EDU positive nuclei) by the total number of cells (DAPI positive nuclei) in each channel.

### 2.11 Live‐cell calcium imaging

To evaluate changes in calcium levels in DC2 cells under various conditions, cells were seeded in a Bioflux microchannel at a density of 4 million/mL and subjected to specific treatments over multiple days. The timeline for each treatment is described as follows: For the conditions named Starvation+Static and Starvation+Shear stress on the first day, cells were seeded into the microchannel. On the second day, standard cell culture media was replaced with a low‐serum media (IMDM supplemented with 1 nM DHT, Fluka, 100 µM MEM nonessential amino acids, 1 mM sodium pyruvate, 50 U/m penicillin G, and 0.5% FBS). On the third day, cells were washed with cell culture media and incubated for 1 h with 5 μM Cal‐520AM dye (ab171868, Abcam) in cell culture media at 33°C. Subsequently, cells were washed with cell culture media for 5 min, rested for 10 min, and exposed to shear stress of 1 dyne/cm^2^ for 5 min or kept under static conditions. For the condition named No starvation+shear stress, serum synchronizing was eliminated as on the first day, cells were seeded into the microchannel, and on the second day, cells were washed with cell culture media and incubated for 1 h with 5 μM Cal‐520AM dye in cell culture media at 33°C, then washed with cell culture media for 5 min, rested for 10 min, and exposed to shear stress of 1 dyne/cm^2^ for 5 min. In all experiments, cells were shielded from bright light after the treatment with calcium dye. For these experiments, images were then acquired as sequential scans every 2 s, with an interval time of 0.7 s. In these analyses, imaging was initiated approximately 5 s before initiating fluid flow under shear stress conditions. Cell boundaries were delineated manually for each capture, and ImageJ was utilized to quantify the mean fluorescence intensity of each cell at every time interval. Each graph depicts the mean ± standard error of the mean (SEM) for six experiments conducted under each condition, with a minimum of 90 cells analyzed per experiment.

To test if the fluid‐shear calcium response in DC2 cells depends on primary cilia, we blocked primary cilia formation either pharmacologically (30 uM Ciliobrevin D for 24 h) or by Ift88 siRNA techniques as previously explained. Following this, the same protocol was employed to introduce Cal‐520AM dye. The cells treated by Ift88 siRNA were fixed after the application of shear stress to determine the frequency and length of primary cilia through labeling with an acetylated tubulin (AC‐Tub) marker.

To identify the source of calcium in shear stress‐induced signaling, Thapsigargin (noncompetitive inhibitor of the Sarco/endoplasmic reticulum Ca ATPase (SERCA)) was used to prevent the release of calcium from internal stores. To determine the role of extracellular calcium in the observed calcium release, cells were subjected to shear stress in a calcium‐free medium, which blocks the entry of external calcium. For SERCA inhibition assay, DMEM (10313039, Gibco) was supplemented with 1 mM Sodium Pyruvate, 4 mM l‐Glutamine (Gibco), 25 mM HEPES (Sigma Aldrich), 10% FBS, and 0.01% DHT (pH: 7.6) in the presence or absence of 1 µM Thapsigargin. Thapsigargin was added to the cells for 15 min before the addition of the calcium probe, followed by a 5‐min washing step. For calcium‐free assays, DMEM with no calcium (21068028, Gibco), was supplemented with 2 mM sodium pyruvate, 4 mM l‐Glutamine, 25 mM HEPES, 10% FBS, 0.01% DHT, and 1 mM ethylene glycol tetraacetic acid (EGTA) (pH: 7.6).

### 2.12 Reverse transcription and quantitative real‐time PCR (RT‐qPCR)

Quantitative PCR (qPCR) was conducted to assess the relative mRNA expression levels. First‐strand complementary DNA of total RNA was synthesized using iScript RT supermix (1725038, Bio‐Rad). Primer sets for qRT‐PCR listed in Supporting Information S1: Table 3 were designed for each gene using the Primer‐Basic Local Alignment Search Tool.

A mixture of 500 nM specific forward and reverse primers and 5 μL SsoAdvanced Universal SYBR Green Supermix (1725270, BioRad) was added to 2 μL cDNA samples. The total volume was adjusted to 10 μL using diethylpyrocarbonate‐treated deionized H_2_O. An RT‐negative control and a no‐template control served as two negative controls. The samples were incubated through the following steps: (a) 5 min at 95°C, (b) 40 cycles of 15 s at 95°C, 15 s at the primer‐specific temperature (between 54°C and 66°C), and 15 s at 72°C, (c) heated from 65°C to 95°C with a rate of temperature change of 0.5°C per 0.5 s. All samples were normalized to housekeeping genes Histh4 and Rps27l. Pfaffl method was employed based on cycle threshold (Ct) comparisons between different samples to calculate fold inductions (Pfaffl, 2001).

### 2.13 Western blot analysis

Cells were lysed using RIPA buffer (150 mM NaCl, 50 mM Tris, 0.1% of SDS, 1% of Triton, 0.5% of deoxycholate, 1 mM EDTA, pH 7.4) supplemented with an anti‐proteinase cocktail (cOmplete, Mini, EDTA‐free Protease Inhibitor Cocktail, Roche). Total protein concentrations were determined utilizing a bicinchoninic acid (BCA) assay (PI23235, Thermo Fisher Scientific). For immunoblotting, 40 µg of protein samples were loaded onto a 10% polyacrylamide gel for SDS‐PAGE. Subsequently, proteins were transferred onto a PVDF membrane. The membranes were then blocked for 1 h with Tris‐buffered saline containing 5% milk and 0.1% Tween 20 (TBST), followed by overnight incubation at 4°C with the primary antibody against IFT88 in 2.5% milk in TBST (Supporting Information S1: Table 1). Following three washes with TBST, the membranes were incubated with the appropriate horseradish peroxidase‐conjugated secondary antibody (Supporting Information S1: Table 2) for 1 h at room temperature. The immunostaining was developed using the Clarity or Clarity Max Western ECL substrate (Bio‐Rad) in the ChemiDoc MP Imaging System (Bio‐Rad). Quantitative analysis was performed by measuring the intensity of band volumes using the ImageJ software and normalizing to β‐actin levels.

### 2.14 Statistical analysis

Statistical analyses were performed using GraphPad Prism version 10.1.2 and data are presented as mean ± SEM. Differences between the two groups were assessed using unpaired *t*‐tests. Comparisons among three or more groups were conducted using two‐way analysis of variance (ANOVA) followed by Tukey's post‐hoc test. A *p*‐value of less than 0.05 was considered statistically significant.

## 3 RESULTS

### 3.1 Primary cilia extend from nonproliferative and differentiated cells of the prepubertal epididymis

Before puberty, the epithelium of the epididymis undergoes significant cell proliferation and differentiation processes, which contribute to the formation of a tubule where various cell types will ensure proper sperm maturation. While primary cilia are sensory organelles contributing to cell proliferation/differentiation and present at the apical surface of epididymal epithelial cells (Bernet et al., 2018), their potential contribution to epididymis post‐natal development remains unknown. Epididymides from 2‐week‐old prepubertal mice were analyzed by confocal microscopy to assess the possible link existing between the presence of primary cilia and ciliated cells' proliferative/differentiated status. At this developmental stage, ARL13B and CENTRIN 2 positive primary cilia were identified at the surface of 40% to 60% of epididymal epithelial cells with a mean length of 3.72 ± 1.46 µm (mean ± SEM), ranging from 0.5 to 13.5 µm. (Figure 1a,b). In addition, we found that primary cilia were exclusively detected in nonproliferating epididymal cells (KI67^−^) (Supporting Information S1: Video 1). To assess whether primary cilia were exclusively present at the surface of undifferentiated cells, epididymides were stained for AQP‐9 principal cell marker, V‐ATPase (subunit A) clear cell marker. Our results indicated that, while V‐ATPase positive clear cells did not exhibit any primary cilia on their surface (Supporting Information S1: Video 2), about 33% of AQP‐9 positive principal cells did expose a primary cilium (Supporting Information S1: Video 3). Additionally, about 30% of undifferentiated ciliated cells were identified. Overall, these findings underscore that in the prepubertal epididymis epithelium, primary cilia exclusively protrude from the surface of non‐proliferative principal cells (Figure 1c).

### 3.2 Fluid shear stress promotes gene expression changes in epididymal principal cells

**Figure 2:**
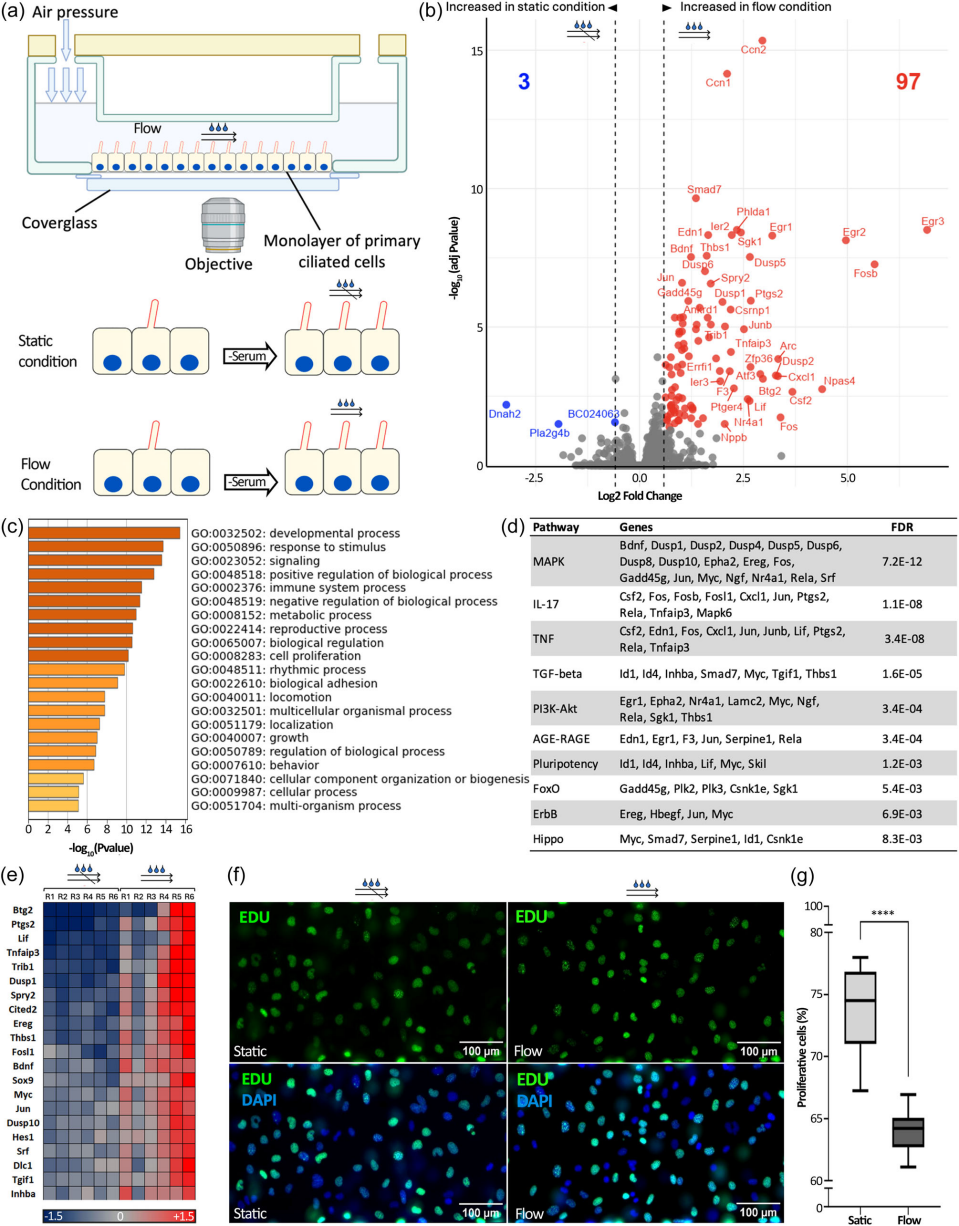
Epididymal principal cells are responsive to fluid shear stress. (a) A schematic of the Bioflux microfluidic system used for cell culture under fluid flow conditions is presented. The system consists of microchannels connecting an inlet well to an outlet, with fluid flow generated by air pressure from a pump. The experimental design involved culturing DC2 cells in microfluidic channels, followed by 48 h of serum starvation to induce primary cilia formation and exposure to either fluid flow or static conditions for 3 h. (b) Volcano plot of gene expression profiling comparing fluid flow and static conditions in DC2 cells. Differential gene expression is presented as log_2_ comparisons of transcripts per million (TPM) values, with upregulated genes in red, downregulated genes in blue (fold regulation> 1.5; P‐adj < 0.05), and nonsignificant changes in gray. (c) Histogram of the main biological processes identified from gene ontology (GO) term enrichment analysis of 143 BPs using algorithms from ShinyGO, DAVID, STRING, Metascape, and MouseMine (Padj < 0.05). (d) Pathway enrichment analysis highlighting pathways significantly influenced by shear stress and the associated genes. (e) Heat map displaying genes associated with cell proliferation. Color range indicates Log_2_FC > 1.5 (upregulated genes); Log_2_FC < –1.5 (downregulated genes). (f) Assessment of proliferative cell rates between static and shear stress conditions over 24 h by EdU incorporation assay. All cells are counterstained with DAPI (blue), and proliferating cells incorporating EdU are detected in green. (g) A significant reduction in EdU incorporation was observed under shear stress conditions. Results are presented as means ± SEM (*n* = 12).

As epididymal ciliated cells come into contact with fluid flow originating from the testis, they are strategically positioned to regulate cellular functions in response to fluid shear stress, similar to what has been observed in the kidney (Kunnen et al., 2018; Mohammed et al., 2018). We established an in vitro system designed to replicate conditions closely resembling physiological in vivo environments to assess epididymal epithelial responsiveness to shear stress. To this aim, we used immortalized DC2 cells that derive from the distal caput principal cells of mice and that form acetylated tubulin (AC‐Tub), and gamma‐tubulin (γ‐Tub) positive primary cilia under low‐serum conditions (Figure 1d), as previously described (Girardet et al., 2022). After exposing DC2 cells to shear stress at 1 dyne/cm², which represents a level relevant to the physiological conditions of epithelial cells (Raghavan et al., 2014), changes in gene expression profiling were observed after shear stress (flow) compared to static (no‐flow) condition (Figure 2a). For instance, 97 DEGs displayed an upregulation exceeding 1.5‐fold change (adjusted *p* value < 0.05), while only 3 DEGs were downregulated (Figure 2b). By mimicking shear conditions met by ciliated epididymal cells at the early stages of post‐natal development, our results underscored the major impact that physical shear stress exerts on the control of epididymal gene expression.

The comprehensive gene ontology (GO) analysis was conducted on these datasets to gain a deeper understanding of the biological processes (BPs) regulated by shear stress‐induced DEGs. In the process of selecting enriched BPs affected by shear stress, a comprehensive approach was used based on different analysis suites, including ShinyGO, DAVID, STRING, Metascap, and MouseMine. Specifically, 23 BPs stand out in all five analyses that exhibited consistent enrichment across all analysis suites and demonstrated significant P‐adj < 0.05 (Supporting Information S1: Table 4). Furthermore, 120 GOs terms consistently emerged from at least 4/5 analyses which brings to 143 BPs potentially affected by flow and the main axes of these BPs are summarized in Figure 2c. The analysis revealed a predominant nuclear localization of DEGs products, with 60% being localized in this subcellular compartment. Clear functional enrichment of DNA binding, regulation of polymerase II, MAP‐Kinase activity, protein‐kinase activity, tyrosine/threonine phosphatase activity, and activity of growth factors were identified, suggesting that shear stress collectively regulates the transcription and translation of genes, influencing the synthesis of proteins and, consequently, various epididymal cell functions.

The exploration of the most representative BPs associated with DEGs revealed regulation of apoptotic processes, vascular development, transcriptional process, and response to growth factors that specifically involve ERK1 and ERK2 cascade, MAPK cascade, mechanical stimulus‐response, inflammatory response, injury response, and drug response. These subprocesses collectively reflect a complex signaling network activated by shear stress and leading to a range of responses associated with MAPK, ERK1, and ERK2 cascades, including cell death, differentiation, and proliferation (Supporting Information S1: Figure 2).

Among signaling pathways that were highly enriched are the MAPK/IL‐17/TGF‐β/and TNF signaling pathways, emphasizing their role in fluid‐flow response (Figure 2d). Overall, this study underscores the complexity of pathway interactions and gene distributions in flow‐induced changes.

### 3.3 Shear stress negatively impacts cell proliferation

The functional enrichment analysis of DEGs revealed that shear stress consistently influences the negative regulation of cell proliferation, a biological function emphasized in all five algorithms used. For instance, Figure 2e illustrates the corresponding heat map, depicting the expression patterns of genes particularly involved in cell proliferation. While these findings support the contribution of shear stress on the regulatory mechanisms governing cell proliferation, this was further investigated through the use of a fluorescent cell proliferation assay under diverse shear conditions. Mechanically induced changes in DC2 proliferation were assessed by subjecting cells to either 1 dyne/cm^2^ or static conditions for 22 h.

**Figure 3:**
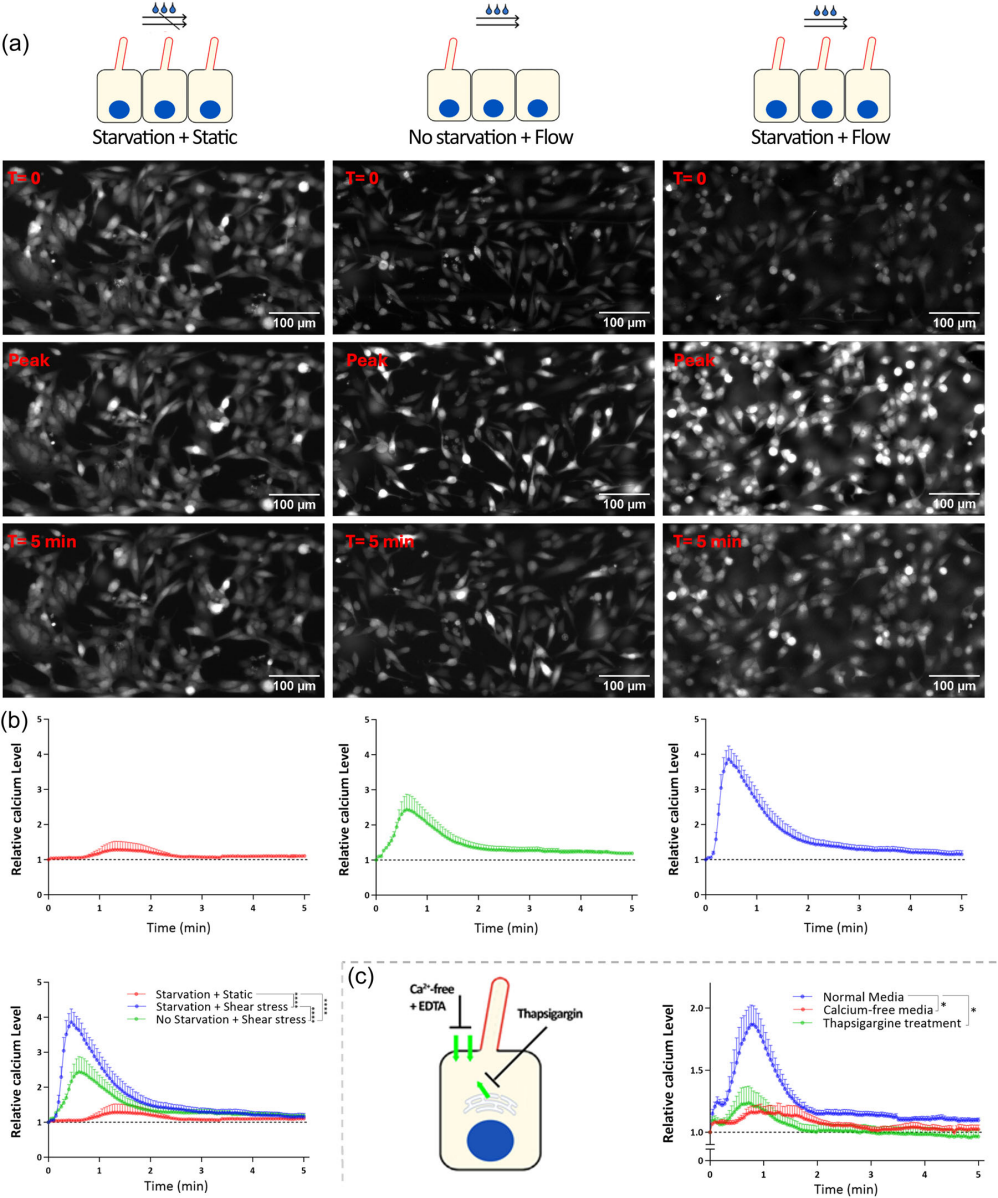
Fluid shear stress induces a rise of intracellular calcium in epididymal principal cells. (a) Time‐lapse imaging of intracellular calcium signals in DC2 cells cultured in BioFlux microfluidic channels under three conditions: Starvation + Static, No Starvation + Flow, and Starvation + Flow where starvation induces primary cilia elongation by arresting the cell cycle in the G0/G1 phase. Calcium probe signaling was acquired at T = 0 (preflow initiation), at the time that the average calcium signal across all cells reached the maximum and 5 min post‐flow initiation. (b) Quantification of relative calcium levels over time (sampled every 3 s for 5 min) under the specified conditions. Data are presented as means ± SEM (*n* = 6). (c) Quantification of intracellular Ca^2+^ signaling in response to fluid shear stress was performed in a control condition, in the presence of the SERCA pump inhibitor, thapsigargin (1 µM), or in the presence of a calcium‐free media containing 1 mM EGTA. Data are expressed as mean ± SEM (*n* = 6).

The Click‐iT EdU Cell proliferation assay was employed to label and quantify proliferating cells. This assay was initially validated by comparing the proliferation rate of DC2 cells in normal culture media versus in low‐serum media, confirming its accuracy (Supporting Information S1: Figure 3). Utilizing the Click‐iT EdU assay, we observed a decrease in the proportion of EdU‐positive proliferative cells from 73.91 ± 0.95 under static conditions to 63.95 ± 0.48 under shear stress conditions (*p* < 0.0001), as shown in Figure 2f,g. These results emphasize the role of shear stress in reducing cell proliferation and are consistent with the negative regulation of the cell proliferation pathways identified through RNA‐seq/in‐silico analyses.

### 3.4 Shear stress augments calcium influx on epididymal cells

Intracellular Ca^2+^ signals as a pivotal second messenger capable of influencing downstream shear stress response, as previously described by Praetorius H.A., et al., from Madin–Darby canine kidney (MDCK) cells (Praetorius & Spring, 2001). To elucidate the impact of fluid shear stress on the elevation of intracellular calcium flux and the source of calcium in this response, a series of experiments were conducted by using a fluorescent calcium probe on epididymal DC2 cells. Under static conditions, a minimal calcium response was observed, with a Ca^2+^ signaling peak close to the baseline (1.28 ± 0.21, arbitrary units(a.u)). Following exposing cells to fluid shear stress of 1 dyne/cm^2^, a rapid increase in intracellular calcium signaling was observed, reaching a peak at 2.44 ± 0.40 a.u. This was observed in the presence of serum, a condition where both nonciliated proliferative cells and resting ciliated cells coexist. Moreover, under serum starvation, a condition that forces cells to enter a quiescent state and stimulate cilia formation, a markedly higher signal was observed, with the peak of Ca^2+^ reaching 3.86 ± 0.34 a.u. This suggests that either the presence of serum itself or an increase in primary cilia frequency potentializes the calcium response to shear stress (Figure 3a,b).

To explore the source of the flow‐induced release of Ca^2+^, thapsigargin, an inhibitor of the sarco/endoplasmic reticulum Ca^2+^ ATPase (SERCA), was employed to deplete intracellular Ca^2+^ stores from the cells. The depletion of intracellular Ca^2+^ stores resulted in a 73% reduction in the peak of Ca^2+^ signaling (from 1.87 ± 0.15 a.u. under normal conditions to 1.23 ± 0.14 a.u. under thapsigargin treatment). Additionally, the influence of extracellular Ca^2+^ was investigated by exposing cells to calcium‐free media supplemented with EGTA (1 mM) in shear stress conditions. Depletion of extracellular Ca^2+^ sources led to an 80% reduction in a peak for Ca^2+^ signaling (1.17 ± 0.06), suggesting that the downstream mechanosensory response is mediated by extracellular calcium influx across the plasma membrane (Figure 3c).

Overall, fluid shear stress significantly increased intracellular calcium signaling in epididymal DC2 cells, particularly in the absence of serum, indicating a potential enhancement of the cells' calcium response by increased cilia frequency. This response involves the release of calcium from intracellular stores triggered by extracellular calcium influx.

### 3.5 Primary cilia mediate a mechano‐sensory response to shear stress

**Figure 4:**
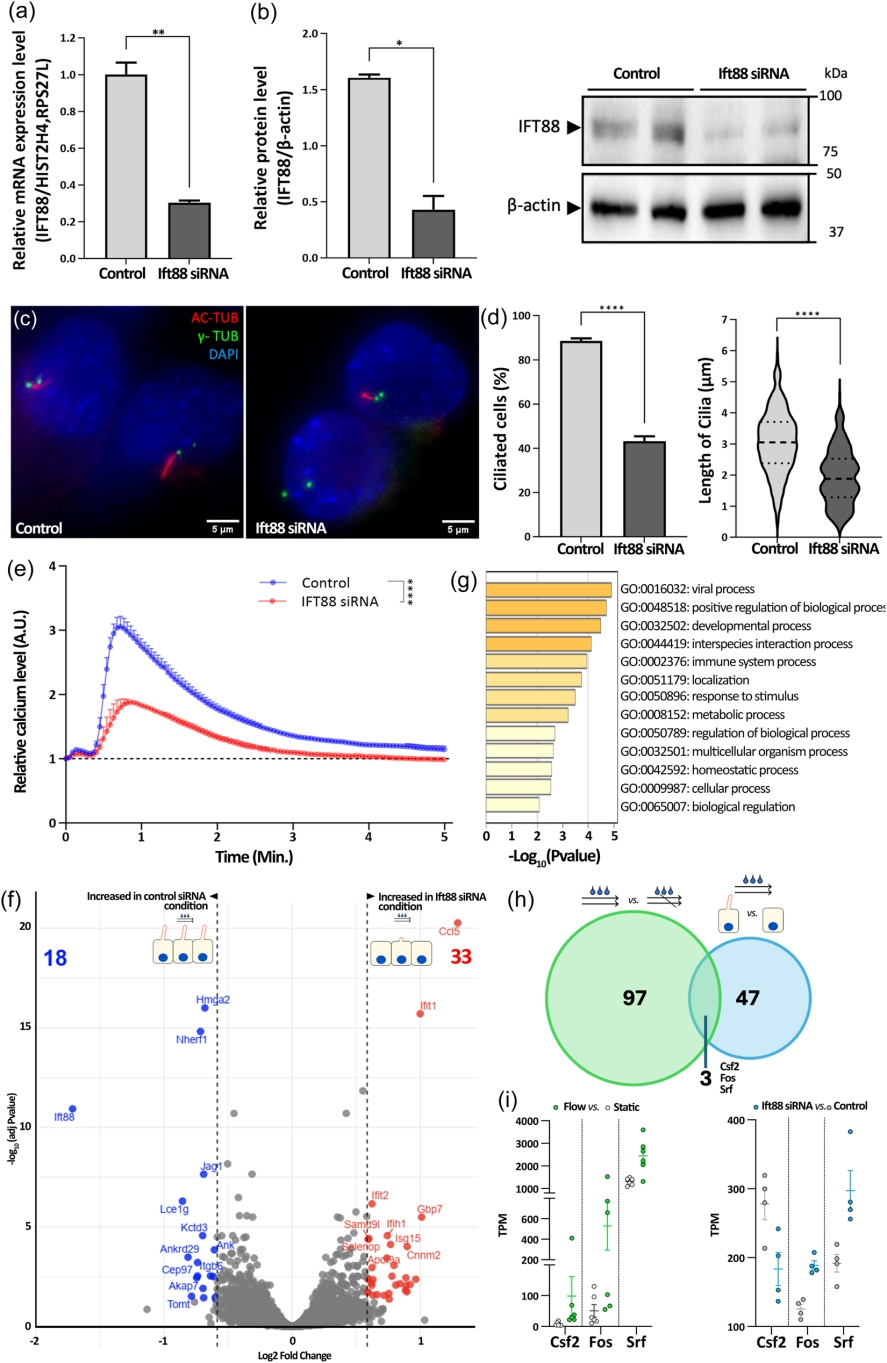
The blockage of primary ciliogenesis initiates alterations in calcium signaling and gene expression under flow conditions. (a,b) The efficiency of Ift88 Knockdown was confirmed by Quantitative PCR and Western blot analyses on cells transfected with either siRNA targeting Ift88 (Ift88 siRNA) or scrambled control siRNAs (Control). (c,d) Examination of ciliary features following staining with AC‐TUB/γ‐TUB indicates a decrease in the proportion of ciliated cells and the detection of shorter cilia in cells transfected with Ift88 siRNA compared to controls (*n* = 3, means ± SEM). (e) Comparative analysis of shear stress‐induced calcium signaling in DC2 cells indicates a significant reduction of calcium signaling in cells transfected with Ift88 siRNA compared to controls (*n* = 3, means ± SEM). (f) Volcano plot illustrating gene expression changes detected by RNA sequencing in DC2 cells transfected with Ift88 siRNA versus controls, underflow condition. (g) Gene Ontology (GO) analysis of the altered transcriptome, highlighting enriched biological functions affected by primary ciliogenesis impairment. (h) Venn diagram illustrating the overlap of specific transcriptional modulations observed in response to fluid flow and Ift88 knockdown conditions. (i) Srf, Csf2, and Fos, are genes both responsive to shear stress and the presence of primary cilia, as illustrated on dot plots.

Recognizing the role of primary cilia as mechanosensors in various biological systems (Nguyen & Jacobs, 2013; Saad Md ZubayerMohieldin et al., 2016; Spasic & Jacobs, 2017), we explored their involvement in modulating the responsiveness of epididymal cells to shear stress. To this aim, the response of DC2 cells to shear stress was assessed following the blockade of primary ciliogenesis through pharmacological and siRNA approaches. Following the pharmacological blockage of primary ciliogenesis by Ciliobrevin D, a reversible and specific inhibitor of cytoplasmic dynein that causes a marked reduction in primary cilia length in DC2 cells (Girardet et al., 2020), we observed a complete loss of the calcium response elicited by shear stress (Supporting Information S1: Figure 4a) as well as significant changes in the expression of some shear‐stress responsive genes, including plasminogen activator inhibitor‐1 (Serpine1) and cellular communication network factor 2 (Ccn2) (Supporting Information S1: Figure 4b).

**Figure 5:**
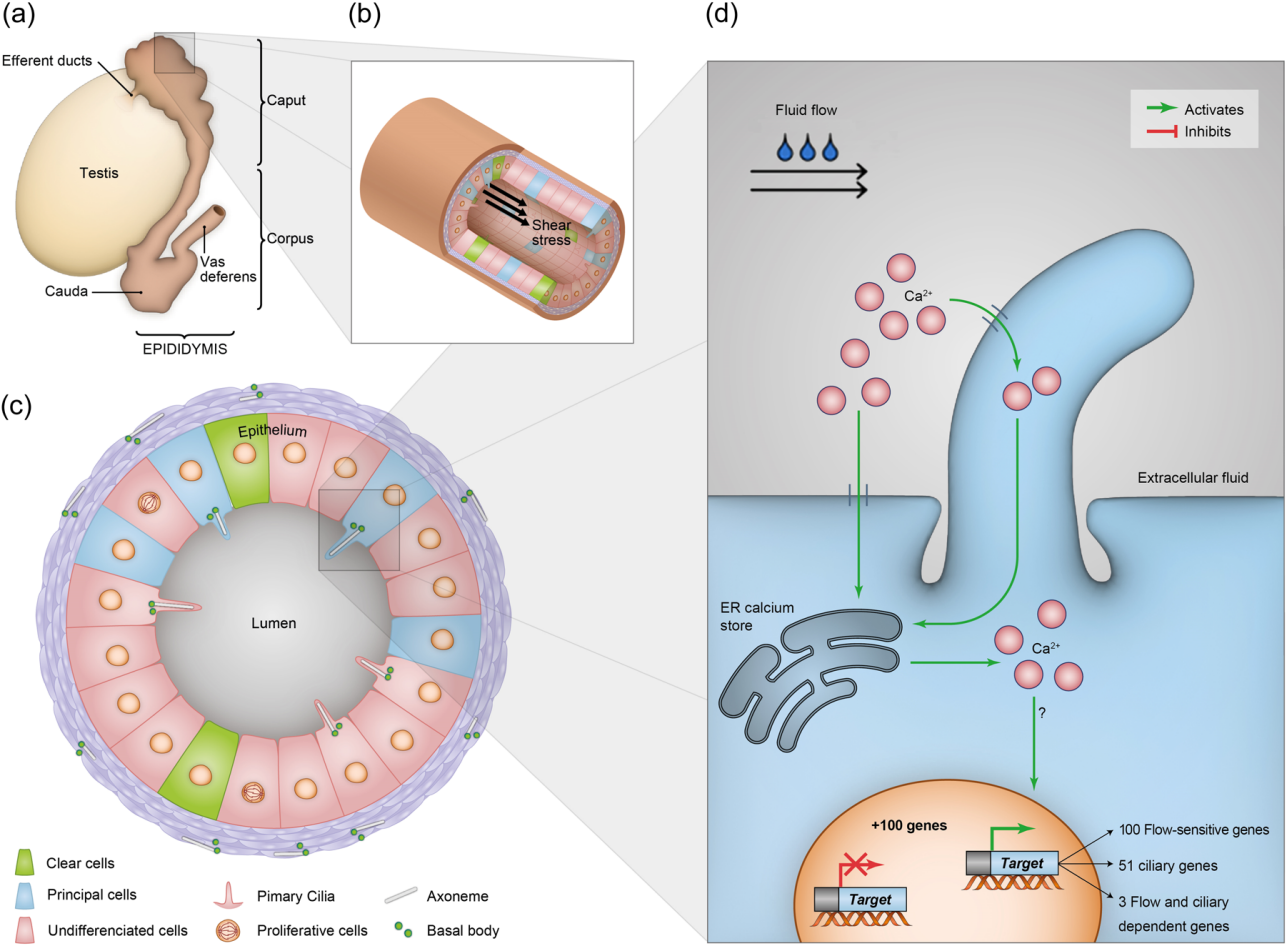
Influence of shear stress on epididymis epithelial cells through primary cilia mechanosensory signaling. (a) Anatomical illustration of the male reproductive system highlighting the epididymis three main segments: caput, corpus, and cauda. (b) Luminal fluid flow‐induced shear stress within the epididymal duct. (c) Cross‐sectional diagram of the epididymal epithelium showing various cell types including undifferentiated cells, differentiated principal, and clear cells. Primary cilia elongate from the basal body of undifferentiated cells and principal cells. (d) Fluid shear stress‐induced calcium signaling and gene expression in principal cells. Fluid flow induces Ca²⁺ influx through the primary cilium, activating the release of calcium from endoplasmic reticulum and leading to the modulation of over 100 genes, including flow‐sensitive genes, cilia‐dependent genes, and genes dependent on both flow and the presence of cilia.

To circumvent the possible off‐target effects of ciliobrevin D, we used a siRNA strategy to prevent the formation of primary cilia by silencing *Ift88*, a gene that encodes a protein essential for ciliogenesis (Taschner et al., 2012). Confirmatory analyses showed that following transfection with Ift88 siRNA (Ift88‐KD), the relative mRNA levels and the relative protein levels were reduced by 3.2 and 3.7 times, respectively, compared to cells transfected with control siRNA (Figure 4a,b). Furthermore, Ift88 knockdown significantly reduced the number of ciliated cells (by 40%) as well as cilia length, as they were on average 1.1 ± 0.07 µm shorter than those formed by control DC2 cells (Figure 4c,d), thus confirming the effectiveness of our silencing strategy (Figure 5).

Blocking primary cilia formation resulted in a 55% reduction in relative calcium signaling. The peak of Ca^2+^ signaling decreased from 3.05 ± 0.16 a.u. in cells treated with control siRNA to 1.92 ± 0.05 a.u. in Ift88‐knockdown cells (Figure 4e), demonstrating that the primary cilium and the associated protein IFT88 play a crucial role in transducing signals, particularly calcium, related to fluid flow‐induced mechanotransduction. To determine the role of primary cilia in the gene expression changes elicited by shear stress, RNA‐seq analysis was conducted on shear stress‐stimulated DC2 cells following Ift88 silencing. The alterations in gene expression were displayed on a volcano plot, contrasting the log_2_‐transformed TPM values of cells for these two conditions. Notably, 33 genes (highlighted as red dots) displayed an upregulation under shear stress, and 18 genes (highlighted as blue dots) demonstrated a downregulation in response to fluid shear stress (FC > 1.5, Padj < 0.05) when primary cilia formation was impaired (Figure 4f). This includes a substantial reduction in the expression of Ift88, providing robust confirmation of the effective knockdown achieved through Ift88 siRNA.

GO enrichment analysis of DEGs revealed a significant enrichment of biological terms, including regulation of cellular component biogenesis and inner ear receptor cell differentiation (Supporting Information S1: Figure 5). TNF signaling, which contributes to the regulation of cell growth, differentiation, and immune response (Larson et al., 2020; Moustakas et al., 2002; Zhang et al., 2017), was highlighted as a major pathway responsive to shear stress in a primary cilia‐dependent manner. Association of this pathway with immune response and the presence of immune system development GO term supports the contribution of primary cilia to immune response, as previously evidenced (Girardet et al., 2020). Additionally, the data set showed relevance to developmental processes such as “morphogenesis of an epithelial sheet” and “immune system development” suggesting a fundamental role of primary cilia in the broad spectrum of cell development and differentiation. To expand on our analysis, broader BPs enriched in our DEG data set were identified. These include biological regulation, response to stimulus, and developmental processes (Figure 4g). These categories cover a wide range of cellular activities, demonstrating the significant impact of primary cilia's mechanosensory functions on cellular behavior. Among DEGs, the expressions of Srf, Csf2, and Fos were modulated under the control of both primary cilia and shear stress (Figure 4h,i), confirming the contribution of epididymal primary cilia in the regulation of target gene expression in response to shear stress.

## 4 DISCUSSION

Luminal flow, which transports fluid from the caput to the distal cauda epididymis, generates fluid shear stress on the surface of epididymal epithelial cells. This suggests the existence of a potential mechano‐sensing signaling on cells that are responsive to luminal fluid flow (Lee et al., 2024). In previous studies, we validated the existence of primary cilia on the surface of the epididymal epithelium (Bernet et al., 2018). These cilia are established structures renowned for their capacity to detect and react to fluid flow in diverse cellular models. The research presented herein provides a thorough investigation into the response of epididymal principal cells to fluid shear stress, with a specific emphasis on the participation of primary cilia in this mechanosensitive process.

### 4.1 The shear stress‐induced response identified in the epididymis mirrors mechanisms found in other biological systems

In the initial phase of elucidating the responsiveness of epididymal cells to fluid shear stress, a comparative analysis was conducted in diverse biofluidic conditions. In the dynamic landscape of gene expression responses to fluid shear stress, we found striking similarities between epididymal cells and renal cells, which represent one of the most extensively studied shear stress‐responsive models (Weinbaum et al., 2010). This underscores a shared mechanism in governing gene expression and cellular functions across various cellular contexts.

Notably, transcriptional changes induced by shear stress in DC2 cells exhibit substantial overlap with those previously reported in kidney epithelial cells. Specifically, 43 out of the 100 DEGs identified were shared among DC2 cells, mouse inner medullary collecting duct‐3 IMCD‐3 cells, and renal proximal tubular epithelial cells PTEC cells (Kunnen et al., 2018; Mohammed et al., 2018), highlighting a considerable level of conservation in the transcriptional responses to fluid shear stress across various epithelial cell types (Supporting Information S1: Figure 6). The list of overlapped DEGs encompasses transcripts involved in fundamental signaling pathways, including TGF‐β (Egr1, Inhba, Thbs1, Tgif1, Fos, Hes1, Lif, Serpine1), TNF (Lif, Edn1, Junb, Ptgs2, Fos), and MAPK (Dusp1, Stk40, Lif, Dusp4, Dusp6, Edn1, Inhba, Mapk6, Errfi1, Ptger4, Epha2, Myc). These pathways play pivotal roles in diverse cellular responses, ranging from the regulation of cell cycle to immune cell homeostasis and stress response.

### 4.2 Shear stress triggers primary cilia‐dependent calcium signaling in epididymal cells

In our study, we observed a rapid increase of intracellular calcium levels in ciliated epididymal DC2 cells subjected to fluid shear stress compared to static conditions. We identified the endoplasmic reticulum (ER) as the source of flow‐induced calcium release. This was confirmed by depleting intracellular calcium stores using thapsigargin, which significantly reduced the calcium signal. Furthermore, depleting extracellular calcium sources resulted in a further reduction in the signal, highlighting the importance of extracellular calcium influx across the plasma membrane in the mechanosensory response to shear stress.

We observed that cells synchronized by low‐serum media show higher Ca^2+^ when exposed to fluid flow in comparison with no starvation condition. We propose two potential explanations for this observation: first, due to synchronization, more cells exhibit primary cilia, implying a direct role of primary cilia in mediating the calcium response; and second, disruption of cell‐to‐cell signaling. Calcium can propagate from one cell to neighboring cells (Praetorius & Spring, 2001), typically through gap junction channels in most epithelia (Clapham, 2007). Although the specific impact of serum starvation on gap junctions in epithelial epididymal cells has not been explored, the absence of a calcium signal in some cells suggests that non‐starved cells may fail to establish connections with neighboring cells, leading to a limited spread of calcium in non‐starved cells within these experimental setups.

The second hypothesis seems more plausible, supported by experiments using Ift88 siRNA transfection. For instance, despite 40% of cells retaining cilia, all cells showed elevated calcium levels, possibly due to heightened intracellular calcium in ciliated cells and its subsequent diffusion to neighboring cells. A blockade of cellular gap junctions would be needed in the future to confirm this hypothesis.

According to our observations, when shear stress was applied to DC2 cells, the calcium signal initially surged and then promptly returned to baseline. As suggested by Munaron, L., et al. this fast and short rise in calcium signal through shear stress stimulation could be principally attributed to the release of calcium from intracellular stores and the activation of SERCA pumps which are most likely triggered by the production of InsP3 (Munaron et al., 2004). This proposition was followed through experimental validation involving the inhibition of SERCA using thapsigargin. The consequential outcome demonstrated a noticeable reduction in the relative calcium signal when subjected to fluid flow conditions. Thapsigargin's inhibitory action on SERCA supports the fact that the calcium signal observed in response to shear stress is closely associated with the release of calcium stores and the regulatory function of SERCA pumps. Furthermore, the flow‐induced release of intracellular calcium is reliant on the presence of extracellular calcium. In other words, calcium influx is a mandatory prerequisite for the occurrence of the signal. This conclusion is drawn from the observation that removing extracellular calcium before the application of the stimulus results in the complete abolition of the calcium signal.

Numerous calcium channels implicated in primary cilia mechanotransduction and present in epididymal epithelial cells are potential participants in this process. For instance, primary cilia contain various calcium‐conducting channels in their membrane, including transient receptor potential (TRP) channels C1/P2/P3/V4 (Nauli et al., 2016). Among these, TRPP2 (also known as Polycystin 2 or PC2) is found in the primary cilia of DC2 cells (Bernet et al., 2018). Additionally, TRPV4 interacts with PC2 to form a mechanosensitive molecular sensor (Köttgen et al., 2008) shown to induce significant potassium (K^+^) secretion and calcium ion influx in rat epididymal epithelial cells (Gao et al., 2022a). This ion movement triggers downstream signaling events, ultimately leading to cellular responses. Piezo1 channels localized on osteocyte primary cilia (Lee et al., 2015), are identified in the rat epididymal epithelium, and activated Piezo1 channels enable Ca^2+^ influx (Gao et al., 2022b). These observations suggest that primary cilia in mouse epididymal epithelial cells respond to fluid shear stress by initiating a calcium‐dependent signaling process that may involve the activation of channels, including TRPV4 or Piezo1, which may in turn regulate the physiological maintenance of the luminal environment in the epididymal duct. Further knock‐down approaches targeting these diverse channel candidates will be needed to examine their contribution to fluid shear stress response in the epididymis.

Furthermore, microvilli are specialized actin‐rich projections on epididymal principal cells that play a key role in the absorption and reabsorption of luminal fluids (Primiani et al., 2007). Stereocilia, similar to microvilli, respond to mechanical forces like luminal flow by bending and triggering the opening of ion channels at their tips (Delling et al. 2016; Fischer 2014). While our study shows that primary cilia partially contribute to mechanosensation in DC2 cells, microvilli may also play a role in mechanotransduction, adding another layer to the mechanobiological response in the epididymis that requires further investigation.

### 4.3 Shear stress is proposed to be an important physical parameter controlling epididymal cell proliferation and differentiation

Based on the mechanism of mechanotransduction of other cell models and according to our result from DC2 epididymal cells, it appears that fluid flow applied at the surface of epididymal epithelial cells triggers a cascade of events that impact various signaling pathways involved in cell cycle progression and proliferation, as well as a rise in intracellular calcium. Calcium is known to play a central role in cell signaling, particularly in regulating cell proliferation (Berridge, 1995; Capiod, 2011; Munaron et al., 2004). Additionally, the calcium signals activate downstream signaling pathways, notably those mediated by MAPK, which are well‐established promoters of cell proliferation (Sun et al., 2015). In the present study, we showed that cells subjected to shear stress conditions exhibited a clear 10% decrease in the rate of proliferative cells, indicating the direct impact of shear stress on cell proliferation dynamics.

The role of primary cilia in the regulation of proliferation and differentiation of epididymal DC2 cells through mechanotransduction is highlighted by the modulation of ciliary gene expression in response to mechanical forces, including the genes Csf2, Srf, and Fos. Colony stimulating factor 2 (Csf2) can influence cell proliferation, as evidenced by studies demonstrating that GM‐CSF (mouse granulocyte‐macrophage colony‐stimulating factor) expression in lung epithelial cells enhances lung growth by promoting the proliferation of type II alveolar cells (Reed et al., 1997). Additionally, GM‐CSF has been shown to play a critical role in facilitating repair processes in response to injury, as observed in a colitis model (Egea et al., 2013). Srf and Fos regulate immediate early genes (IEGs), which are quickly and transiently activated in response to various stimuli and participate in the initial cellular response to growth factors (Greenberg & Ziff, 1984; Norman et al., 1988). Specifically, Srf binds to the serum response element (SRE) in the Fos gene promoter, triggering its rapid induction (Esnault et al., 2014). This interaction controls cell cycle regulation, apoptosis, cell growth, and differentiation, emphasizing the significance of primary cilia in cellular mechanosensory processes of epididymal cells. Additional evidence supporting this idea comes from the discovery that proliferative cells in the 14‐day‐old mouse caput epididymis do not possess primary cilia. This indicates a potential link between shear stress, primary cilia, and the control of cell proliferation in the epididymis. This finding aligns with the understanding that primary cilia are reabsorbed during the G1/S and M phases of the cell cycle (Mill et al., 2023). None of the differentiated clear cells and only some of the differentiated principal cells in the 14‐day‐old mouse epididymis possessed primary cilia. Although DC2 cells are immortalized and represent differentiated principal cells from the adult mouse epididymis, they extend primary cilia that come into contact with the intraluminal compartment, similar to what is observed during the prepubertal stage (Bernet et al., 2018). While the shear stress response identified in these in vitro studies suggests a role for primary cilia in cell proliferation/differentiation through shear stress signaling, additional in vivo models are required to determine its contribution to the overall post‐natal development of the epididymis.

### 4.4 Shear‐stress induced response can influence sperm surrounding fluid environment

In this study, we observed that shear stress has a significant impact on epididymal DC2 cells, as indicated by the identification of DEGs encoding secreted protein, including Csf2, Cxcl1, Ccn2, Lif, Hbegf, Serpine1, Sdn1, Ereg, Thbs1, Adamts1, Bdnf, Gadd45g, and Plaur. Considering the epididymis' primary role in maturing and storing spermatozoa, with sperm gaining motility in the proximal parts and maturation being influenced by the local environment provided by the epididymal fluid, the mechanisms that control the expression of these secreted proteins is particularly noteworthy.

Among the secretory proteins identified, plasminogen activator inhibitor‐1 (Serpine1), ADAM metallopeptidase with thrombospondin type 1 motif 1 (ADAMTS1), and leukemia inhibitory factor (LIF) are notable for their roles in sperm development and fertility. Serpine1 is important for sperm capacitation and motility, enhancing the sperm's fertilization ability (Liu, 2007). ADAMTS1 contributes to testicular development and sperm maturation, facilitating sperm‐egg interactions by promoting adhesion to the zona pellucida (Aydos et al., 2016; Hernández‐Delgado et al., 2023). LIF enhances sperm motility and survival, playing a role in sperm transport and viability within the female reproductive tract (Attar et al., 2003). The upregulation of these genes by shear stress underscores the significance of mechanical cues in modulating epididymal functions and sperm maturation. Understanding the mechanotransduction pathways involved in regulating these genes particularly relevant to sperm maturation could provide valuable insights into how mechanical forces influence the acquisition of male fertility.

## AUTHOR CONTRIBUTIONS

Sepideh Fakhari conducted most of the experiments and analysis on mouse tissues and analysis on DC2 cells and drafted the manuscript. Farnaz Asayesh, Laura Girardet, and Gabriel Campolina‐Silva contributed to the experiments. Denis Soulet, Gabriel Campolina‐Silva, Marie‐PierScott‐Boyer, and Arnaud Droit contributed to data analysis. Clémence Belleannée, Jesse Greener, and Sepideh Fakhari contributed to the study design and manuscript preparation. Clémence Belleannée, Sepideh Fakhari, and Gabriel Campolina‐Silva contributed to data interpretation and manuscript editions. All authors have approved the final version of this article.

## CONFLICT OF INTEREST STATEMENT

The authors declare no conflict of interest.

## Supporting information

Supporting information.

Supporting information.

Supporting information.

Supporting information.
