# Supplementary material for: Shear stress effects on epididymal epithelial cell via primary cilia mechanosensory signaling
Source: J Cell Physiol. 2024 Nov 7;240(1):e31475. doi: 10.1002/jcp.31475 (PMC11733861; doi:10.1002/jcp.31475)

# Supplementary Tables.

**Supplementary Table 1**. Information regarding primary antibodies used in this study

| **Type** | **Name** | **Host species** | **Dilution WB** | **Dilution IF** | **Company** | **Catalog number** |
| --- | --- | --- | --- | --- | --- | --- |
| Monoclonal | Acetylated tubulin | Mouse | N/A | 1-400 | Sigma | T7451 |
| Monoclonal | β-actin | Mouse | 1-5000 | N/A | Sigma | A5441 |
| Monoclonal | Gamma Tubulin | Mouse | N/A | 1-400 | Abcam | ab 27074 |
| Polyclonal | V-ATPase | Rabbit | N/A | 1-3000 | Gifted by Breton, S.^1,2^ | - |
| Monoclonal | EpCAM | Rabbit | N/A | 1-400 | Proteintech | 324207 |
| Polyclonal | Aquaporin 9 | Rabbit | N/A | 1–100 | Alpha diagnostic | AQP91-A |
| Polyclonal | Ift88 | Rabbit | 1-2000 | 1-100 | Proteintech | 13967-1-AP |
| Monoclonal | Ki67 | Rat | N/A | 1/1000 | eBioscience | 14-5698-82 |

1. Paunescu, T. G. *et al.* Expression of the 56-kDa B2 subunit isoform of the vacuolar H+-ATPase in proton-secreting cells of the kidney and epididymis. *American Journal of Physiology-Cell Physiology* **287**, C149–C162 (2004).

2. Brown, D., Paunescu, T. G., Breton, S. & Marshansky, V. Regulation of the V-ATPase in kidney epithelial cells: dual role in acid–base homeostasis and vesicle trafficking. *Journal of Experimental Biology* **212**, 1762–1772 (2009).

**Supplementary Table 2**: Information regarding secondary antibodies used in this study

| **Type** | **Species** | **Dilution** | **Company** |
| --- | --- | --- | --- |
| Fluorescence 647 | Goat anti-Rabbit | 1-400 | Jackson |
| Fluorescence 647 | Donkey anti-Rat | 1-400 | Jackson |
| Fluorescence 568 | Goat anti-Mouse | 1-400 | Invitrogen |
| Fluorescence 488 | Goat anti-Mouse | 1-400 | Invitrogen |
| HRP | Donkey anti-Rabbit | 1-10000 | Jackson |

**Supplementary Table 3**: Information regarding primers used for real-time PCR analysis used in this study

| **Gene name** | **Primer sequence, 5'->3'** | | **Efficiency** | **Optimal temperature** |
| --- | --- | --- | --- | --- |
|  | *Forward* | *Reverse* |  |  |
| Plk2 | GAACCTCATGGATGGTGGTGA | CACCTGAAATGTGCCGTCAT | 107.4 | 64°c |
| Serpine1 | CATGTTTAGTGCAACCCTGGC | GGCTGAGATGACAAAGGCTGT | 102.4 | 64°c |
| Ccn1 | CTTTTCAACCCTCTGCACGC | CTCGTGTGGAGATGCCAGTT | 105.7 | 64°c |
| Ccn2 | GCCTACCGACTGGAAGACAC | GTAACTCGGGTGGAGATGCC | 126 | 64°c |
| Hist2h4 | TCATCTACGAGGA GACCCGT | GOGTACACCACATCCATAGC | 102 | 66°c |
| Rps27l | TCGAAAGOGAGCAGTTCGTC | CAGATCTCTAGCCAGGGGCA | 108 | 66°c |

**Supplementary Table 4:** Consistently Enriched Biological Processes by shear stress-induced DEGs: Shared findings across all multiple analysis tools (P-adj <0.05)

| Term description | # Genes | FDR |
| --- | --- | --- |
| positive regulation of cell death | 22 | 2.55E-10 |
| positive regulation of apoptotic process | 21 | 3.01E-10 |
| cellular response to transforming growth factor beta stimulus | 12 | 3.23E-10 |
| negative regulation of cell cycle | 17 | 1.07E-09 |
| negative regulation of cell population proliferation | 21 | 1.07E-09 |
| skeletal muscle cell differentiation | 9 | 1.99E-09 |
| blood vessel morphogenesis | 17 | 1.99E-09 |
| rhythmic process | 14 | 9.89E-09 |
| positive regulation of cell migration | 17 | 3.33E-08 |
| inactivation of MAPK activity | 6 | 4.01E-08 |
| negative regulation of ERK1 and ERK2 cascade | 8 | 1.41E-07 |
| positive regulation of smooth muscle cell proliferation | 9 | 1.47E-07 |
| circadian rhythm | 9 | 2.63E-06 |
| leukocyte differentiation | 12 | 4.31E-06 |
| heart development | 15 | 4.36E-06 |
| angiogenesis | 11 | 9.74E-06 |
| ERK1 and ERK2 cascade | 5 | 1.01E-05 |
| MAPK cascade | 8 | 4.69E-05 |
| peptidyl-tyrosine dephosphorylation | 6 | 0.00013 |
| positive regulation of pri-miRNA transcription by RNA polymerase II | 4 | 0.00023 |
| cellular response to fibroblast growth factor stimulus | 5 | 0.00031 |
| peptidyl-threonine dephosphorylation | 3 | 0.00039 |
| fat cell differentiation | 5 | 0.0026 |

# Supplementary Figures

**Supplementary Figure 1:** Efficiency tests showing the impact of varying small interfering RNA (siRNA) concentrations (10-200 nM) on IFT88 silencing in DC2 cells. The optimal concentration of 100 nM was selected for subsequent experiments based on the highest.

**
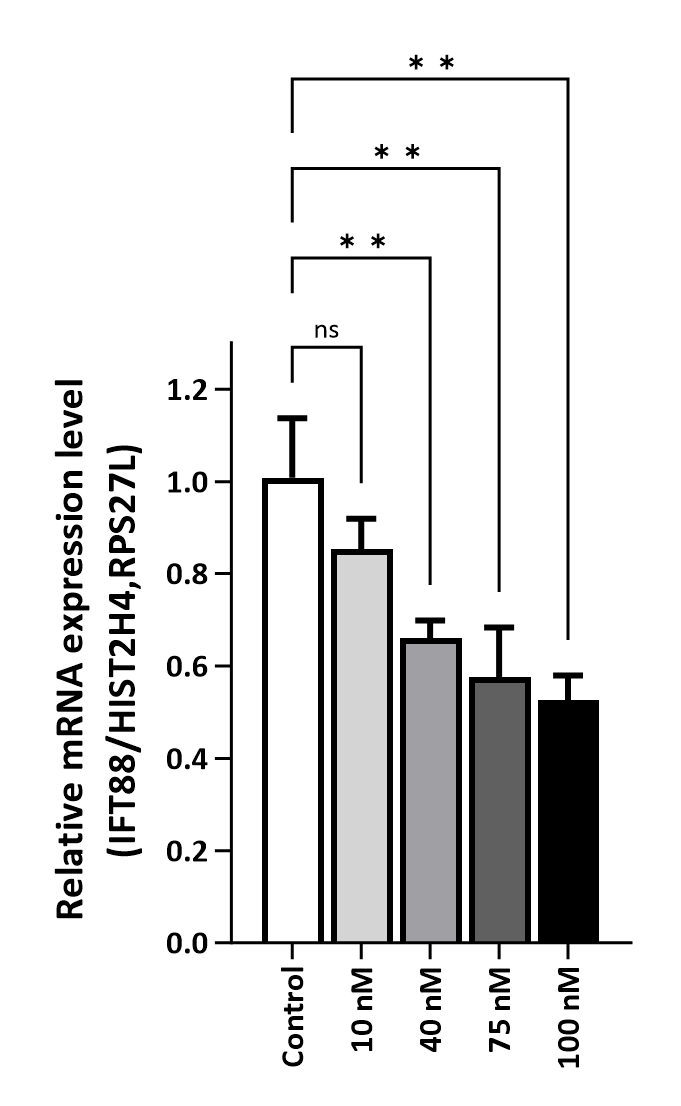
**

**Supplementary Figure 2:** Scatter plot illustrating cluster representatives after semantic redundancy reduction. Terms are grouped in a two-dimensional space using multidimensional scaling based on the semantic similarities of GO terms. Cluster 'representatives' are highlighted in red, while unique terms are in yellow. The color of each bubble represents the p-value associated with the GO term, and the size indicates the term's frequency in the Mus Musculus GO database.


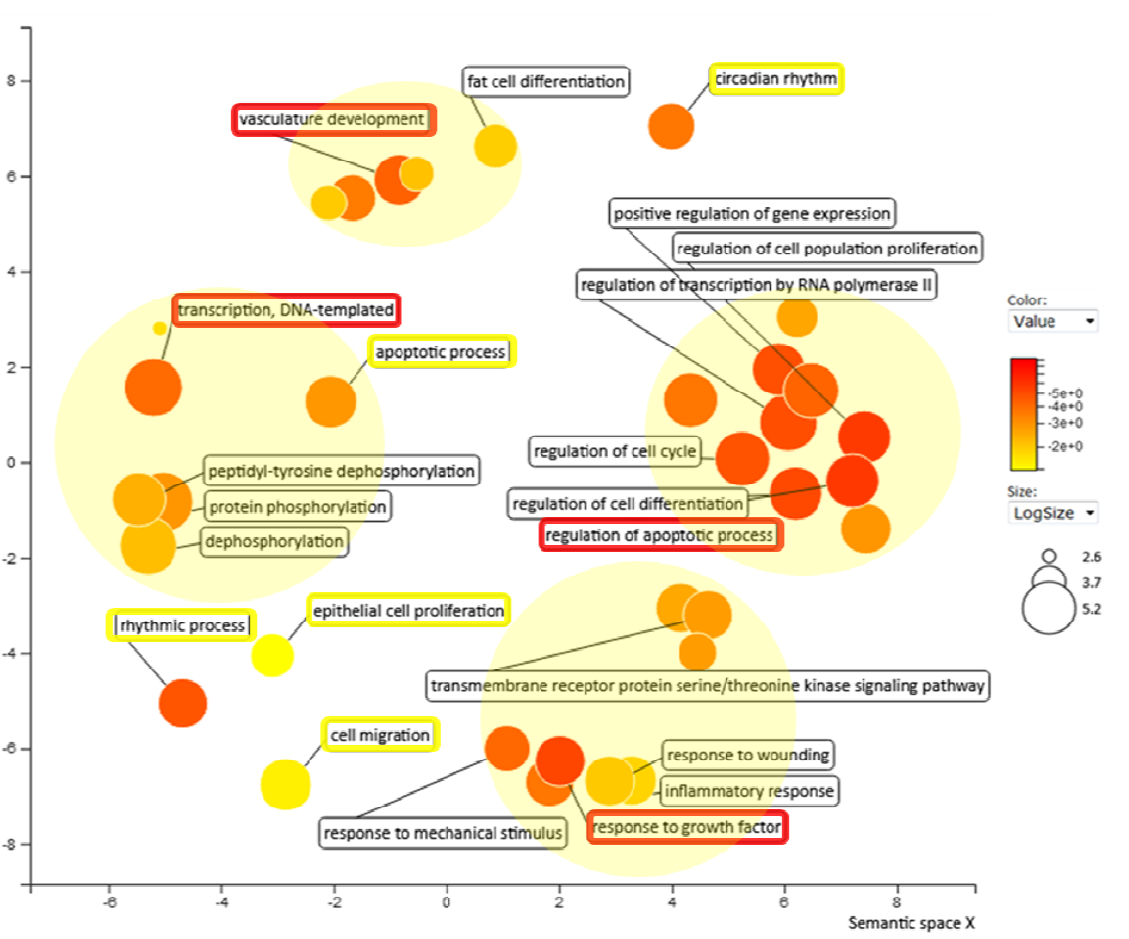


**Supplementary Figure 3:**

**
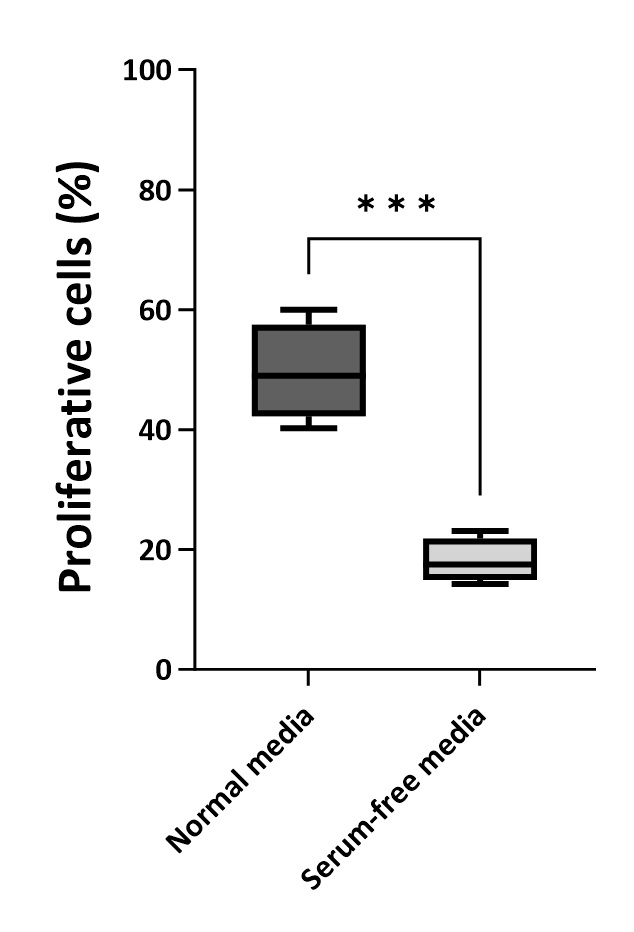
**

**Supplementary Figure 4** Pharmacological blockade of primary ciliogenesis in DC2 cells using Ciliobrevin D abolishes the calcium response to shear stress(A), and alters the expression of shear-stress responsive genes (B), providing scientific evidence for the critical role of primary cilia in shear stress mechanosensing.


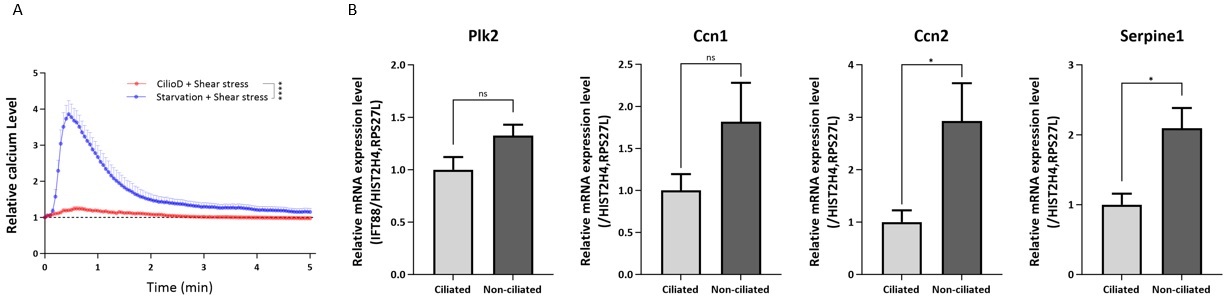


**Supplementary Figure 5:** GO term-enrichment analysis, histogram showing the top significant GO terms associated with DEGs in IFT88 siRNA vs Control.

**
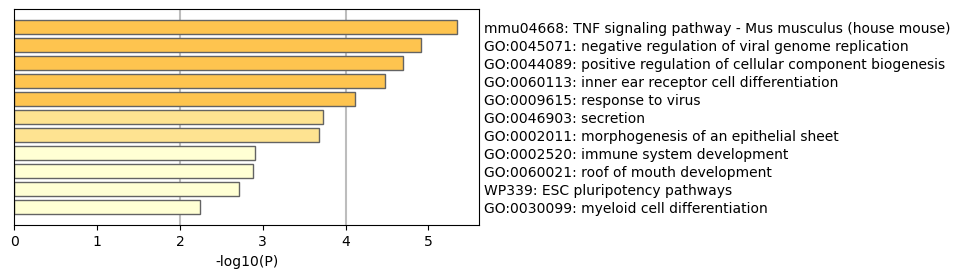
**

**Supplementary Figure 6:** The overlap analysis of the 100 DEGs in DC2 cells with genes DEGs under fluid shear stress conditions in two renal cell types, Proximal Tubular Epithelial Cells (PTECs) and Inner Medullary Collecting Duct-3 (IMCD3) cells, based on literature and the list of 43 overlapped DEGs.

**
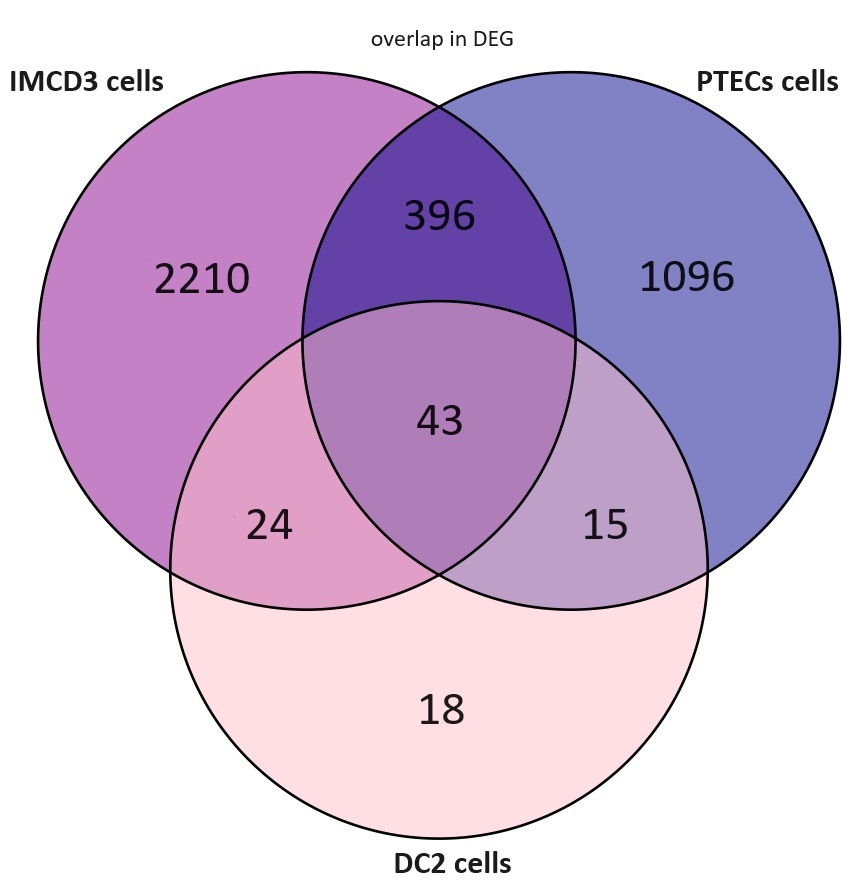
**

# GSEA_GO analysis:

In this study, we aimed to comprehensively investigate the genome-wide effects of fluid shear stress on DC2 epididymal cells. To achieve this, we subjected DC2 cells to fluid shear stress flow (1 dyn/cm²) for 3 hours prior to collecting cellular extracts for RNA sequencing experiments. DESeq2 analysis revealed significant differential expression of 106 protein-coding genes (DEGs; cut-off: adjusted p-value < 0.05; Log2 fold change >0.5 or <-0.5) in response to shear stress.

Gene Set Enrichment Analysis (GSEA) was conducted to comprehend the scope of transcriptional changes induced by fluid shear stress in DC2 cells. As anticipated, a plethora of GO terms exhibited significant enrichment when comparing DC2 cells subjected to fluid shear stress versus static conditions. This encompassed 461 terms in "Biological Process," 71 in "Molecular Function," and 43 in "Cellular Component" categories. Consequently, the Top-15 activated (depicted by blue bars) and the Top-15 repressed GO terms (red bars) from each category were meticulously ranked based on their enrichment score and visually presented in three figures.


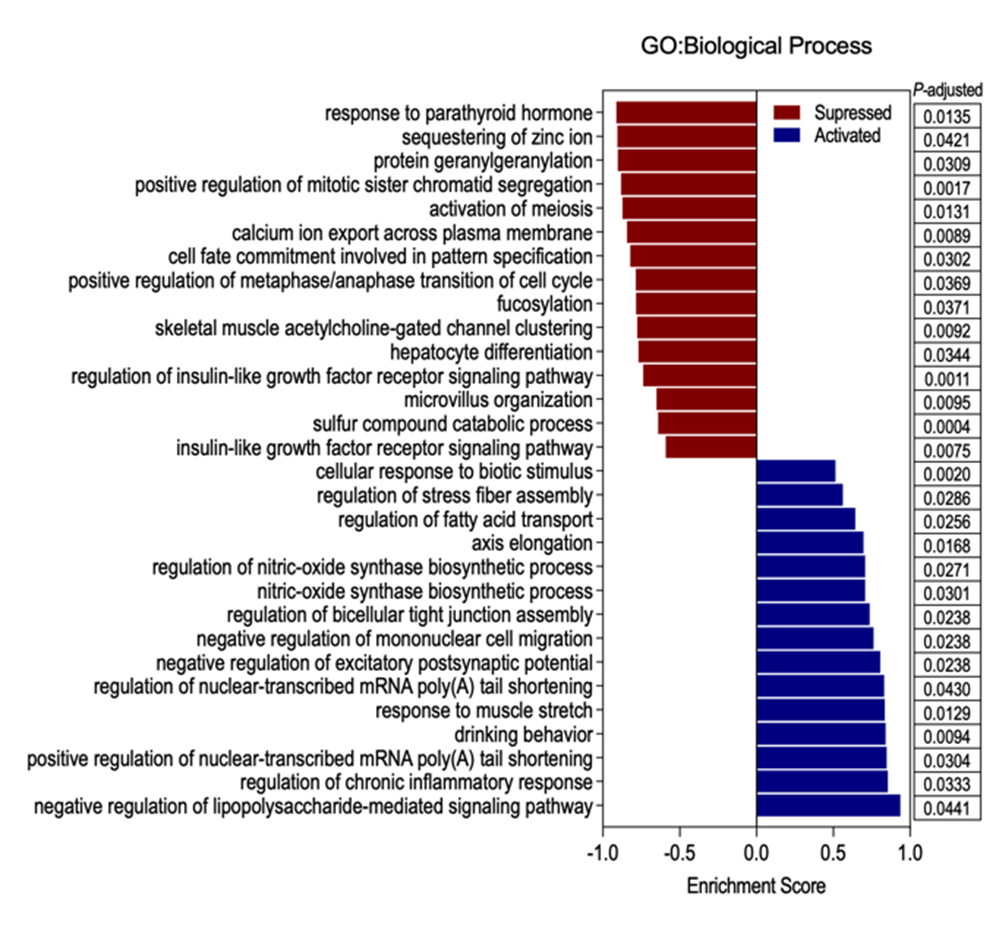


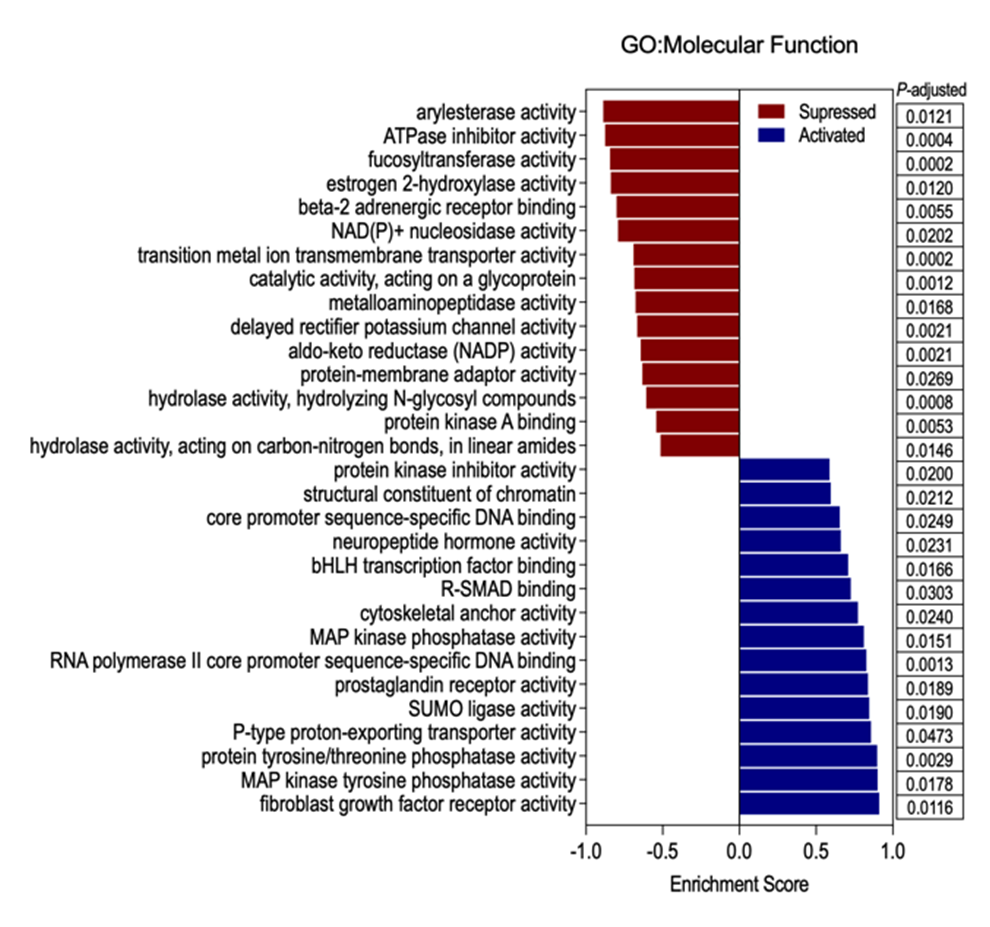


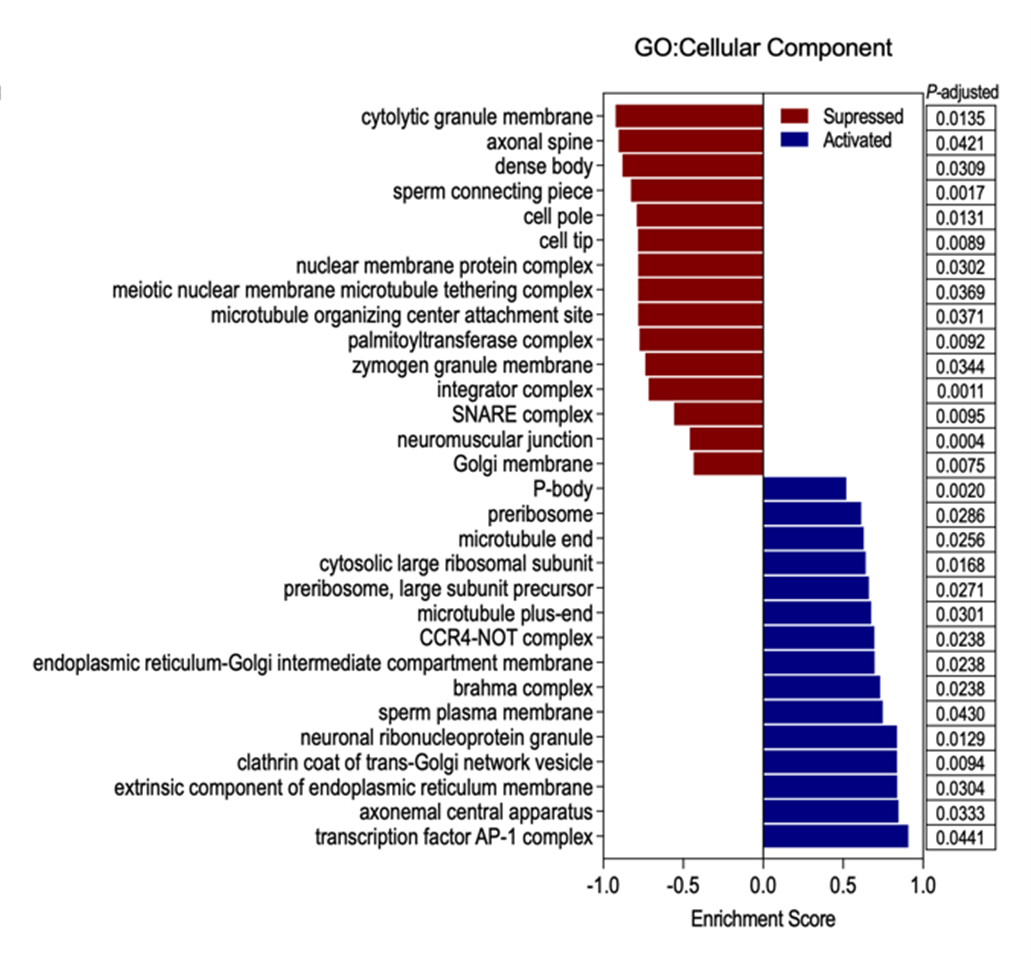

Supplement: Supplementary file 1 — Supporting information. [file JCP-240-0-s002.docx]
